# Remarks on the Case of Miss M'Avoy

**Published:** 1824-02

**Authors:** Thomas Renwick


### ART. V.—*Remarks on the Case of Miss M'Avoy.* By THOMAS RENWICK, M.D.

THE interest the former editors of your valuable Journal appeared to take in the case of the late Miss M'Avoy, and the circumstance that it is the only publication in which the various opinions are fully and impartially detailed to a certain period, induce me to make it the vehicle of transmitting to the public the contents of this letter.

The narrative and continuation of her case have been given to the public, and the statements therein made, and the symptoms described by me, her attending physician, have been allowed by various witnesses, who, independent of me and of each other, visited and examined her, to be correct and true. I can only, therefore, refer the obloquy under which this cruelly and unjustly aspersed female suffered during the latter part of her life, to feelings which do no credit to the persons who could, without remorse, and without proof of any kind, but of the most negative nature, and in opposition to positive facts, assert she was an impostor.

If the public wish to be informed upon the subject, they may peruse the books containing all the arguments in favour of and

---

against her. I shall only observe, that I should not have resumed this subject, if it had not been that the editors of different works have ventured to assert that she was an impostor, and to declare her supporters to be weak and credulous. This assertion would, of course, go into the world as matter of fact, if not rebutted; and I therefore take the liberty of stating, that those who have had the best opportunity of judging of her state of health, and of her powers, deny the assumption they have made, and think they hazarded their own credit by asserting that, from the report of prejudiced individuals, which, if they had read the account as detailed in the narrative and continuation of the case, would have induced them to have given a very different opinion. Scepticism is useful in society, but the acknowledgment of error is the first step in the advancement of science, and I hope yet to see those editors follow this golden rule. If she were not blind, the modes of blindfolding were of that nature as, in my opinion, to have completely effected the object during the different trials. If, however, the public, after a fair trial, think them not efficient, I must concede to their opinion; but I must say it is then impossible to blindfold any one. I do not, however, rest upon this point alone, because I firmly believe she was blind from the period mentioned in the Narrative to that of her death. I shall, therefore, merely recapitulate some of those facts which still induce me to hold the opinion I have advanced.

I have proved in the Narrative that Miss M'Avoy, when I was first called in, laboured under those symptoms generally attendant upon approaching blindness, previous to her becoming blind. That blindness had taken place, independent of her own assertion, was proved by the pupils not being affected by the sudden passing of the hand or fingers across the eyes, nor by the sudden approach of a lighted candle. It was still more confirmed by the common actions of a person seeing being changed into those usually observed in a person suddenly affected with total blindness. The convulsions with which she was seized in a few days after my first visit, were presumptive proofs of the brain, or its appendages, suffering under disease; and, from the appearances upon the arachnoid membrane, and in the fourth ventricle of the brain, as described by Mr. Harrison, the respectable anatomist of Dublin, who visited her twice before her death, and who afterwards inspected the body, it was more than probable effusion had taken place. From the fluid obtained being similar to that effused upon serous surfaces, it is probable inflammation was the cause of this accumulation, by which the brain was compressed, and from which it was relieved by the discharge. At this time it might have been fairly urged by her opponents, if the fluid were derived from the brain, and had caused blindness, that the discharge, by removing the compression upon the brain, and upon the optic nerves, supposing them to have been affected by the compression, might have restored her to sight. This effect, however, did not take place after this or any other of the discharges of a similar nature; for she could bear the sudden approach of a lighted candle to her eyes without flinching; and, when the naked eyeballs were exposed to the vivid rays of the sun, they were not affected by the intensity of its light; nor did she wink or close the eyelids, nor did tears flow. Could any one who was not blind be subjected to similar experiments without instantly complaining? Indeed, almost every attainable mean has been used to ascertain if she possessed sight in any degree; but, instead of the different experiments establishing this fact, they have uniformly proved decisive of her blindness.

If we combine these facts with the general appearance of the eyes, the dilatation of the pupils at first, and their inability to contract,—the irregularity of their motions afterwards, when they occasionally contracted or were dilated,—the effect which the application of pointed and other instruments to the pupil, across and over the cornea, produced,—the insensibility to the sudden presentation of a pistol, demonstrative of the inert state of the optic nerves,—no one ought to assert she was not blind.

If to these proofs we add the appearances upon dissection of the base of the brain, it will cause the sceptical to pause before they deliver an opinion upon a subject of this nature, until they have maturely considered it; and, although many of the symptoms mentioned in the Narrative and Continuation of Miss M'Avoy's case were not accounted for by the appearances upon dissection, yet enough was seen to show that disease had existed, and had been relieved by the means employed.

The nervous system is of so complicated a nature, varied so much in its sympathies, and with which we are so little acquainted, that we cannot be surprised to find the most accurate dissection lead to no reasonable conclusion. We sometimes see diseases apparently attacking different organs of the human body, which, upon examination, seem never to have affected them; and the cause of death is often found in a remote part, where no symptoms indicated disease. The more expert the anatomist, the more cautious is he of deciding from symptoms alone, and the physiologist has often to deplore the construction of a beautiful theory, which the knife dashes to the ground.

In examining the base of the brain, (Mr. Harrison says,) and the course of the cerebral nerves, the olfactory appeared healthy: behind these, the union of the optic nerves on the sphenoid bone was seen, smaller than usual, very thin, and flaccid, as if the nervous substance did not fill the investing membrane, a little pale fluid being between the nerve and its tunic. The nerve also, from this to the orbit, appeared very thin and flat, more particularly the left, which was peculiarly flaccid and delicate: traced back to their connexion with the thalami optici and tubercula quadragemina, they appeared, as usual, broad and flat. The pituitary gland and infundibulum were of natural situation. and all the cerebral nerves presented a healthy appearance, having their usual attachments to the brain, and taking their usual course. All other parts on the base of the brain appeared healthy; and the same remark passed on looking on the divided surface of the spinal marrow, which was cut in the cervical region. On opening the orbits, we observed them to contain much loose and watery adeps; the muscles of the eyeballs were very weak and pale; the nerves were all distinct, and the optic appeared of the usual size and consistence. On cutting across both these nerves, we perceived the red spot, pointing out the situation of the arteria centralis retinæ, so commonly seen in this incision. On dissecting the eyeball itself, all the parts were distinctly seen in this beautiful organ, perfectly free from disease. The lens and its surrounding transparent fluids, the retina and its investing tunics, with the pigmentum nigrum, were satisfactorily seen in both eyes. On the retina, the punctum aureum was very brilliant. We could not see any of the branches of the before-mentioned artery, either on the retina or piercing the vitreous humour. The more than usual quantity of adeps might be accounted for from the circumstance, that there was generally a predisposition in this subject to the accumulation of fat, which might have arisen from the quiescent state in which the body was kept, by her inability to move about. Its loose and watery appearance might have been caused by the diminished excitement which a state of blindness would naturally produce. Had sight existed, and the motions of the different parts of the eye been properly exercised, this superfluity would have been necessarily removed by the quicker action of the absorbents upon active organs.

I think it unnecessary to dwell upon this subject longer. I have done my duty as the medical attendant of Miss M'Avoy, and as a member of the public.

*Liverpool; November 17th*, 1823.

