# Erratum

**Published:** 1824-03

**Authors:** 


ERRATUM.

In our last No. p. 114, for "we perceived," read "we did not perceive."

